# DNA demethylation is associated with malignant progression of lower-grade gliomas

**DOI:** 10.1038/s41598-019-38510-0

**Published:** 2019-02-13

**Authors:** Masashi Nomura, Kuniaki Saito, Koki Aihara, Genta Nagae, Shogo Yamamoto, Kenji Tatsuno, Hiroki Ueda, Shiro Fukuda, Takayoshi Umeda, Shota Tanaka, Shunsaku Takayanagi, Ryohei Otani, Takahide Nejo, Taijun Hana, Satoshi Takahashi, Yosuke Kitagawa, Mayu Omata, Fumi Higuchi, Taishi Nakamura, Yoshihiro Muragaki, Yoshitaka Narita, Motoo Nagane, Ryo Nishikawa, Keisuke Ueki, Nobuhito Saito, Hiroyuki Aburatani, Akitake Mukasa

**Affiliations:** 1 Department of Neurosurgery, Graduate School of Medicine, The University of Tokyo, 7-3-1 Hongo, Bunkyo-ku, Tokyo 113-8655 Japan; 2 Genome Science Division, Research Center for Advanced Science and Technology, The University of Tokyo, 4-6-1 Komaba, Meguro-ku, Tokyo 153-8904 Japan; 3 Department of Neurosurgery, Kyorin University Faculty of Medicine, 6-20-2 Shinkawa, Mitaka-shi, Tokyo 181-8611 Japan; 4 Department of Neurosurgery, Cancer and Infectious Disease Center Tokyo Metropolitan Komagome Hospital, 3-18-22 Honkomagome, Bunkyo-ku, Tokyo 113-8677 Japan; 5 Department of Neurosurgery, Dokkyo Medical University, 880 Kitakobayashi, Mibu-machi, Shimotsuga-gun, Tochigi 321-0293 Japan; 6 Department of Neurosurgery, Graduate School of Medicine, Yokohama City University, 3-9 Fukuura, Kanazawa-ku, Yokohama 236-0004 Japan; 7 Department of Neurosurgery, Tokyo Women’s Medical University, 8-1 Kawada-cho, Shinjuku-ku, Tokyo 162-8666 Japan; 8 Department of Neurosurgery and Neuro-Oncology, National Cancer Center Hospital, 5-1-1 Tsukiji, Chuo-ku, Tokyo 104-0045 Japan; 9 Department of Neuro-Oncology/Neurosurgery, Saitama Medical University International Medical Center, 1397-1 Yamane, Hidaka-shi, Saitama 350-1298 Japan; 10 Department of Neurosurgery, Graduate School of Medical Sciences, Kumamoto University, 1-1-1 Honjo, Chuo-ku, Kumamoto, 860-8556 Japan

**Keywords:** Cancer genomics, CNS cancer, DNA methylation

## Abstract

To elucidate the mechanisms of malignant progression of lower-grade glioma, molecular profiling using methylation array, whole-exome sequencing, and RNA sequencing was performed for 122, 36 and 31 gliomas, respectively. This cohort included 24 matched pairs of initial lower-grade gliomas and recurrent tumors, most of which showed malignant progression. Nearly half of IDH-mutant glioblastomas that had progressed from lower-grade gliomas exhibited characteristic partial DNA demethylation in previously methylated genomic regions of their corresponding initial tumors, which had the glioma CpG island methylator phenotype (G-CIMP). In these glioblastomas, cell cycle-related genes, RB and PI3K-AKT pathway genes were frequently altered. Notably, late-replicating domain was significantly enriched in the demethylated regions that were mostly located in non-regulatory regions, suggesting that the loss of DNA methylation during malignant transformation may involve mainly passive demethylation due to a delay in maintenance of methylation during accelerated cell division. Nonetheless, a limited number of genes including *IGF2BP3*, which potentially drives cell proliferation, were presumed to be upregulated due to demethylation of their promoter. Our data indicated that demethylation of the G-CIMP profile found in a subset of recurrent gliomas reflects accelerated cell divisions accompanied by malignant transformation. Oncogenic genes activated by such epigenetic change represent potential therapeutic targets.

## Introduction

Glioma is the most common type of primary malignant brain tumor in adults. Gliomas are classified as World Health Organization (WHO) classification I to IV based on histological malignancy^1^. Glioblastoma (GBM) is categorized as the most malignant type (grade IV) and is associated with a median survival of approximately 15 months despite intensive multimodal treatment. On the other hand, diffuse astrocytoma and oligodendroglioma, both of which are categorized as grade II, grow slower than GBMs. Nevertheless, grade II gliomas recur frequently and are difficult to control over a long time period. Indeed, more than half of recurrences of diffuse astrocytomas manifest as malignant progression^2,3^. A population-based study showed that the mean period of progression from lower-grade glioma to GBM was 5.3 years and from anaplastic astrocytoma to GBM was 1.4 years^4^. These observations indicate that lower-grade glioma is a fatal disease largely because this tumor type is prone to recur with malignant progression.

Recent advances in genetic analyses of gliomas have identified key alterations involved in gliomagenesis, such as *IDH* mutation^5^. *IDH* mutation is common in low-grade gliomas and is considered to be an early event that persists during progression to secondary GBM^6,7^. In contrast, *IDH* mutations are rare in *de novo* GBM, which is also called primary GBM. Genome-wide methylation analyses showed that the glioma CpG island methylator phenotype (G-CIMP), which is named after the well-known CIMP status observed in colorectal cancers, is frequent in lower-grade gliomas and secondary GBMs and is tightly associated with the presence of *IDH* mutations^8,9^. In lower-grade tumors, *IDH* mutation causes accumulation of 2-hydroxyglutarate, leading to inhibition of the enzymatic activity of ten-eleven translocation 2 (TET2), which plays an important role in DNA demethylation^10,11^. As a consequence, DNA hypermethylation occurs globally in those tumors, resulting in the phenotype called G-CIMP. Such epigenetic alterations contribute to gliomagenesis.

Because most patients with lower-grade glioma eventually die due to malignant transformation of the initial tumor, detailed understanding of the molecular mechanisms that promote malignant progression is important for determining strategies to control disease and develop novel therapeutic strategies. To date, molecular analyses have demonstrated that disruption of the RB pathway, loss of *CDKN2A*, amplification of *CDK4* or *CDK6*, and loss of heterozygosity at 10q or *PTEN* mutation are frequent events during malignant progression of lower-grade gliomas^12^. Furthermore, rapidly advancing technologies of omics analysis are now providing a tremendous opportunity to further understand the molecular mechanisms involved in complicated processes such as tumor progression. Indeed, recent analysis using next-generation sequencing (NGS) and microarray technology has revealed dramatic changes including complicated subclonal somatic mutations and branched evolution of the glioma genome, as well as loss of DNA hypermethylation in G-CIMP tumors coincident with upregulation of cell cycle-related genes during malignant progression of lower-grade gliomas^13–15^. Despite accelerated research in this field, the mechanisms associated with lower-grade gliomas have not been clarified, partly because of difficulties in obtaining paired initial tumor tissue and recurrent tissue samples.

Here we analyzed molecular alterations during malignant progression of lower-grade gliomas using our own large sample that included 24 paired samples of primary and recurrent tumors. Integrated analysis of this large data set demonstrated characteristic changes in the molecular profile and identified several key molecules involved in malignant transformation.

## Results

### DNA methylation profile analysis of gliomas grouped according to their biological characteristics

**Figure 1:**
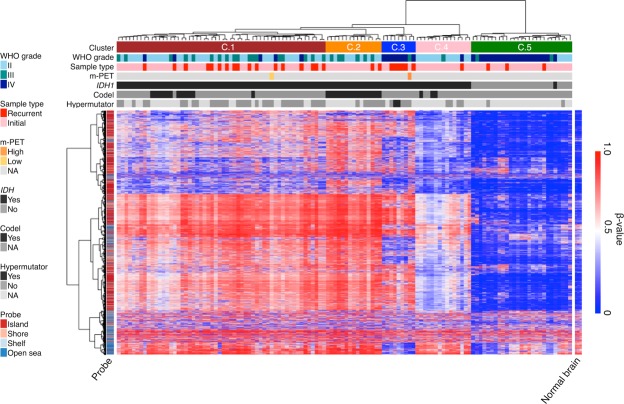
DNA methylation profile of 122 gliomas. Heatmap of the DNA methylation profile of 122 gliomas of this study. Unsupervised clustering for 122 tumors was performed using the top 10,000 variant probes. Heatmap of three normal brain samples using the same probes is shown on the right. The CpG island status of each probe is shown on the left. Sample information is shown at the top. *m-PET* ^11^C-methionine positron emission tomography, *NA* not available, *codel* 1p/19q codeletion.

Methylation profiling was performed for 122 gliomas including 24 pairs of primary and recurrent tumors using the Infinium HumanMethylation450K BeadChip (Supplementary Table S1). Unsupervised clustering analysis grouped these gliomas into five clusters, which we called Cluster 1 (C.1) to C.5 in this study (Fig. 1). Each cluster somewhat represented glioma histology and genetics.

All but one IDH-mutant glioma showed genome-wide DNA hypermethylation as previously reported^9,16^ and were grouped in C.1, C.2, C.3, or C.4, whereas all IDH-wildtype tumors were clustered in C.5. Among IDH-mutant gliomas, typical DNA hypermethylation called G-CIMP was observed in tumors in the C.1 and C.2 clusters. C.1 mostly consisted of astrocytic tumors that do not have the 1p/19q codeletion (codel), whereas all gliomas in C.2 were oligodendroglial tumors with the codel. Although the clustering analysis using only IDH-mutant samples (C.1–C.4) still could not clearly distinguish codel from non-codel tumors (Supplementary Fig. S1a), the futher analysis, using only C.1 and C.2 samples, did so, indicating that astrocytomas and oligodendrogliomas have their own characteristic methylation profile (Supplementary Fig. S1b).

In contrast to C.1 and C.2, C.3 gliomas had common demethylated DNA regions compared to typical G-CIMP tumors and formed a subgroup within G-CIMP clusters (C.1–C.4). We considered that the C.3 subgroup was similar to what was reported as “G-CIMP-low,” whereas the C.1 subgroup was similar to “G-CIMP-high” in The Cancer Genome Atlas (TCGA) study^16^. Notably, most C.3 tumors (8/9 cases) were IDH-mutant GBMs (grade IV), including six recurrent tumors and two *de novo* GBMs, which are generally considered to have progressed from lower-grade astrocytomas (Supplementary Table S2). In contrast, tumors that recurred as grade II or III remained in the C.1 G-CIMP group. No tumor in the C.3 cluster was an oligodendroglial tumor with the codel, suggesting that this characteristic demethylation preferentially occurs in IDH-mutant astrocytic tumors. Gliomas in C.4, most of which were grade II, also showed decreased methylation compared to tumors in C.1 or C.2; however, unlike C.3 tumors, the methylation profile of C.4 tumors was similar to the normal brain pattern shown on the right side of the cluster in Fig. 1, suggesting that decreased methylation in C.4 tumors was due to considerable contamination of normal tissues in C.4 tumors.

This data set included two samples that were obtained from different regions of one IDH-mutant non-codel glioma. Among them, one grade III sample from the core malignant region, which showed high uptake with ^11^C-methionine positron emission tomography (m-PET), was clustered in C.3, whereas the other grade II sample from the peripheral region, which showed low uptake with m-PET, was clustered in C.1. This case, with intratumoral heterogeneity, also suggested that demethylation preferentially occurs in the course of malignant progression of IDH-mutant astrocytic tumors.

### The characteristics of DNA demethylation in the G-CIMP-demethylated tumors

To explore the characteristics of the methylation changes that were possibly associated with malignant transformation of IDH-mutant astrocytic tumors, comparative analysis was performed between typical G-CIMP astrocytic tumors without the codel in the C.1 cluster (C.1 non-codel gliomas; n = 44) and G-CIMP-demethylated tumors (C.3 gliomas; n = 9). The C.1 tumors with the codel (oligodendrocytic tumors) were excluded from this analysis. As expected, C.3 gliomas were more hypomethylated than C.1 non-codel gliomas (Supplementary Fig. S2a). Of 419,382 examined probes, 33,695 probes were significantly hypomethylated (*q*-value < 0.05 and methylation difference > 0.2) in C.3 tumors, whereas only 635 probes were hypermethylated. Significantly hypomethylated probes in C.3 tumors were most frequently located in “Open sea”, whereas hypermethylated probes were in “Island”, as defined by the UCSC annotation (Supplementary Fig. S2b). Thus, most demethylated regions in C.3 tumors were located outside CpG islands.

### Relationship between replication timing and DNA methylation status

**Figure 2:**
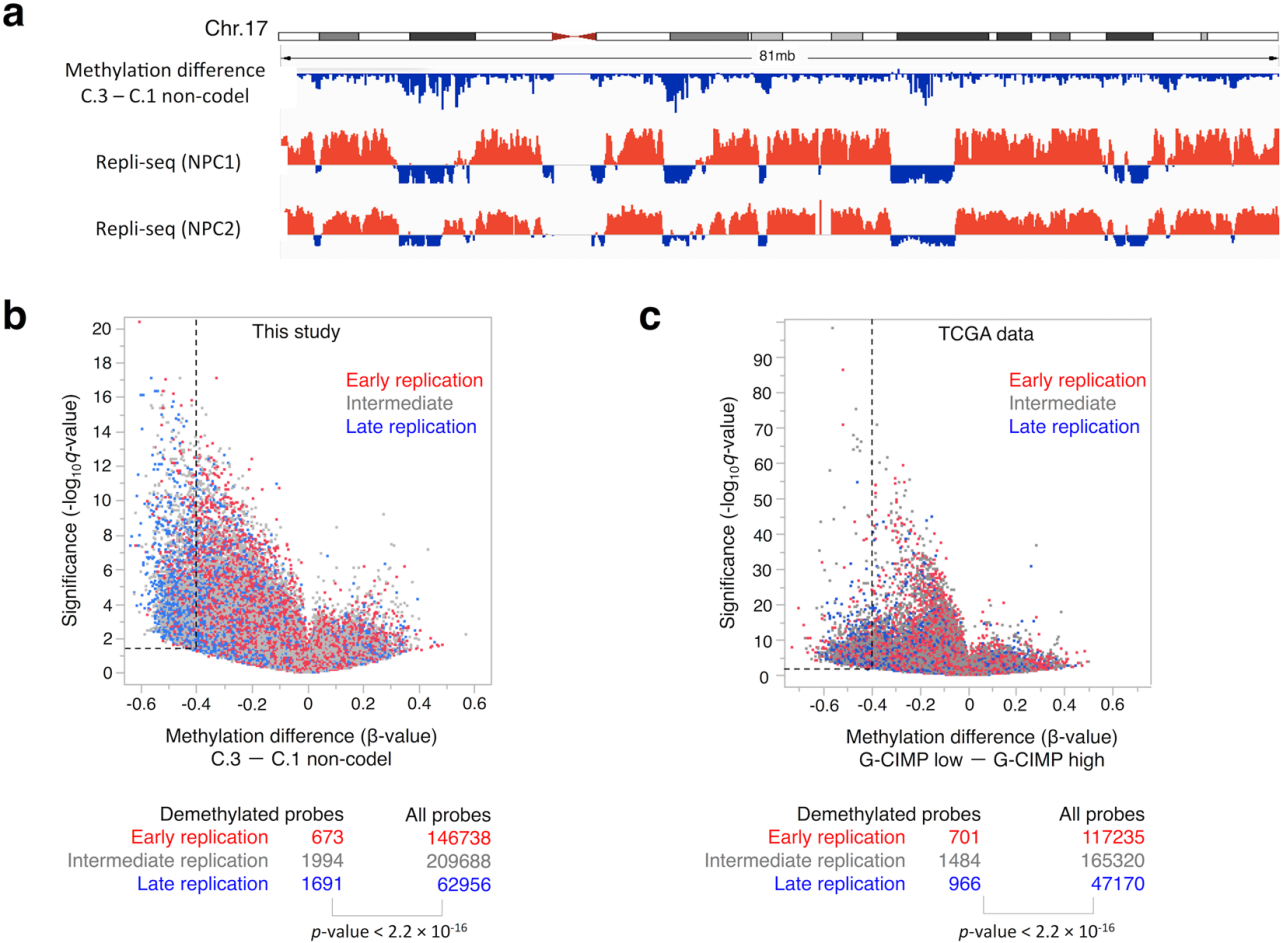
Methylation changes related to replication timing in IDH-mutant gliomas. (**a**) Correlation between replication timing data of Repli-seq for two NPCs and demethylated regions in G-CIMP-demethylated (C.3) tumors during glioma progression along human chromosome (chr.) 17. Red and blue bars of replication timing data indicate positive (early) and negative (late) replication timing values, respectively. (**b**) A volcano plot comparing the β-value of each probe between our G-CIMP-demethylated (C.3) and C.1 non-codel tumors. One dot represents one probe and is colored according to the replication timing at the position of each probe. The *q*-values were calculated using a paired two-sided moderated Welch’s *t*-test and the Benjamini-Hochberg method. Probes were considered to be significantly demethylated in C.3 tumors when the *q*-value was <0.05 and the methylation difference <−0.4. The distribution of significantly demethylated probes in C.3 tumors and all probes according to replication timing was statistically analyzed using the Chi-square test. (**c)** An analysis using G-CIMP-low and -high tumors from the TCGA study was performed in the same way in b.

Because recent studies comparing the DNA methylation level between tumors and normal tissue demonstrated that most cancer-associated hypomethylated regions occurred in late-replicating chromatin domains in rapidly dividing cells^17,18^, we hypothesized that demethylated regions of G-CIMP tumors may be associated with late-replicating chromatin domains. Therefore, we performed comparative analysis of our methylation data for G-CIMP-demethylated (C.3) and C.1 non-codel tumors with genome-wide replication timing profile data of neural progenitor cells (NPCs) from a previous report^19^. As we expected, the comparative schema along chromosome demonstrated a remarkable correlation between the demethylated regions and the late replicating areas shown in blue bar (Fig. 2a). The genome-wide evaluation also showed that probes in the late-replicating areas were significantly enriched in the demethylated (*q*-value < 0.05 and methylation difference > 0.4) regions (*p*-value < 2.2 × 10^−16^, Chi-square test) (Fig. 2b). This finding was verified using TCGA data by comparing replication timing and the methylation difference between the G-CIMP-high and G-CIMP-low groups (*p*-value < 2.2 × 10^−16^) (Fig. 2c)^16^.

The previous report also showed that in cell lines, DNA methylation levels gradually decreased with increasing population doublings and that the rate of methylation loss was higher in late-replicating regions in later population doublings^20^. These data together suggested that acceleration of the cell cycle in malignant tumor cells may lead to preferential demethylation in late-replicating regions. A recent study showed that cell lines established from IDH-mutant gliomas often lose the *IDH1* mutant allele and that these cell lines without the *IDH* mutant allele exhibit a demethylation profile similar to progressed IDH-mutant tumor tissue^21^. Interestingly, however, our examination using previously reported data from two IDH1-mutant cell lines that possess both the *IDH* mutant and wildtype alleles^22^ revealed that these cell lines were also grouped together with our G-CIMP-demethylated (C.3) tumors by clustering analysis (Supplementary Fig. S3). Thus, we considered that IDH1-mutant cell lines, which usually grow much faster than the original tumor in the brain, may lose methylation preferentially in late-replicating regions and show a similar methylation profile as recurrent glioma samples.

### Difference in gene expression changes in G-CIMP-demethylated tumors

**Figure 3:**
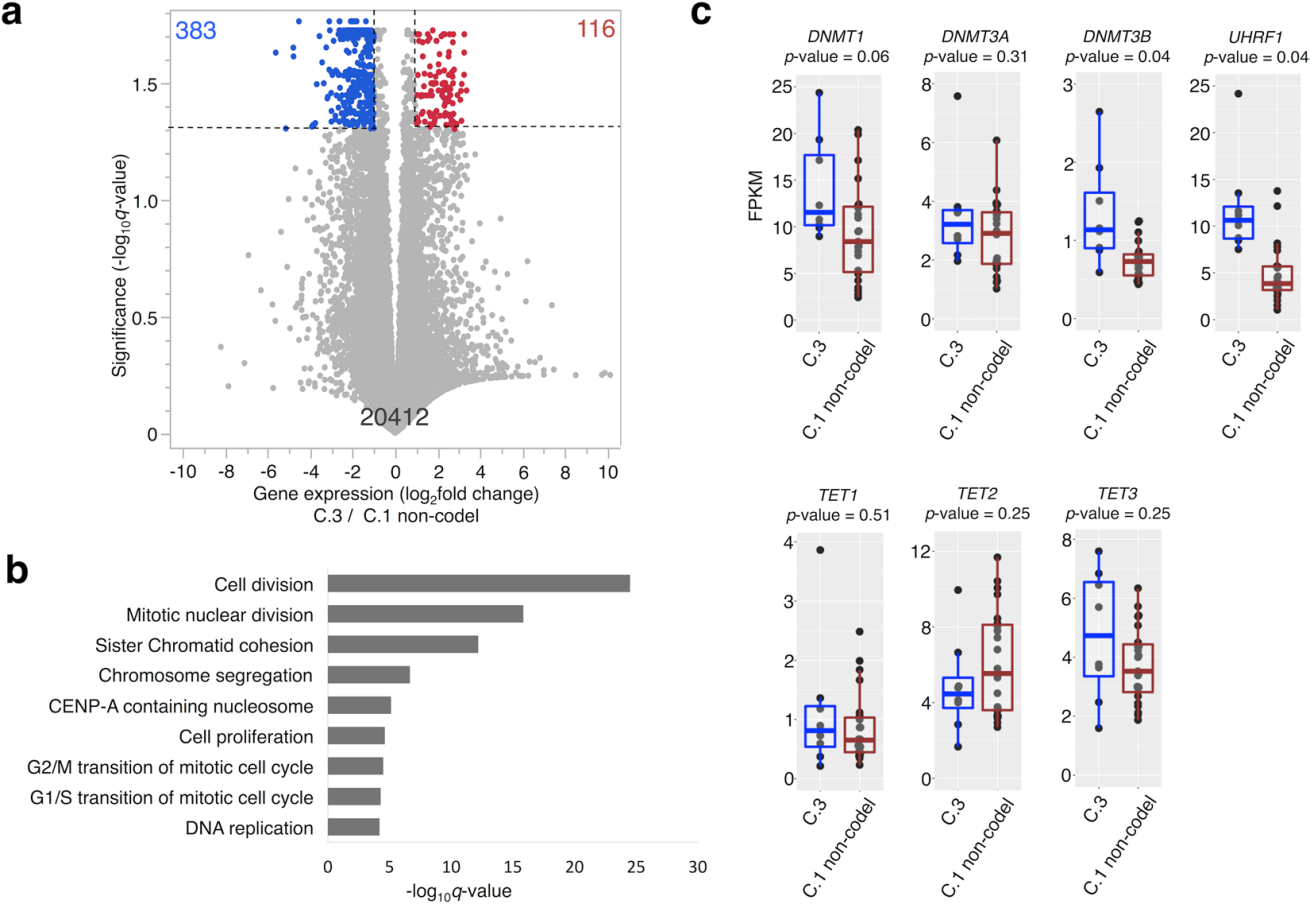
Gene expression comparison between G-CIMP-demethylated (C.3) and C.1 non-codel tumors. (**a**) A volcano plot comparing gene expression between C.3 and C.1 non-codel tumors is shown. One dot represents one gene. The *q*-values were calculated using a paired two-sided moderated Welch’s *t*-test and the Benjamini-Hochberg method. Genes were considered to be significantly different when the *q*-value was <0.05 and the fold change was >2. Significantly upregulated genes are colored red, whereas significantly downregulated genes are colored blue. (**b**) The top nine functional enrichments of significantly upregulated genes in C.3 are shown. (**c**) Box plots of gene expression (*DNMT1*, *DNMT3A*, *DNMT3B*, *UHRF1*, *TET1*, and *TET2*) comparing C.3 and C.1 non-codel tumors are shown. The *p*-values were calculated using a paired two-sided moderated Welch’s *t*-test.

Next, we compared the gene expression level between G-CIMP-demethylated (C.3) (n = 8) and C.1 non-codel tumors (n = 23) using RNA sequencing (RNA-seq) data. One hundred sixteen genes were upregulated (*q*-value < 0.05; Welch’s *t*-test and Benjamini-Hochberg method and the fragments per kilobase of exon per million mapped reads (FPKM) fold change >2), and 383 genes were downregulated (*q*-value < 0.05 and FPKM fold change <0.5) (Fig. 3a, Supplementary Table S3). Gene ontology analysis for differentially expressed genes with the database for annotation, visualization, and integrated discovery (DAVID, http://david.abcc.ncifcrf.gov/) demonstrated that upregulated genes were enriched in cell division, mitotic nuclear division, and chromatin segregation (Fig. 3b). This finding suggests that acceleration of cell division in C.3 tumors possibly led to the loss of DNA methylation in late-replicating domains.

Although not statistically significant in a multiple comparison, *DNA methyltransferase 1* (*DNMT1*), *DNMT3B*, and *Ubiquitin-like PHD* and *RING finger domain-containing protein (UHRF1)*, which contribute to maintenance of methylation activity of DNMT1, were rather slightly upregulated in G-CIMP-demethylated (C.3) tumors (Fig. 3c). In contrast, *TET1* and *TET2*, which are related to active DNA demethylation, were not upregulated in C.3 tumors.

### Upregulated genes with promoter demethylation

**Figure 4:**
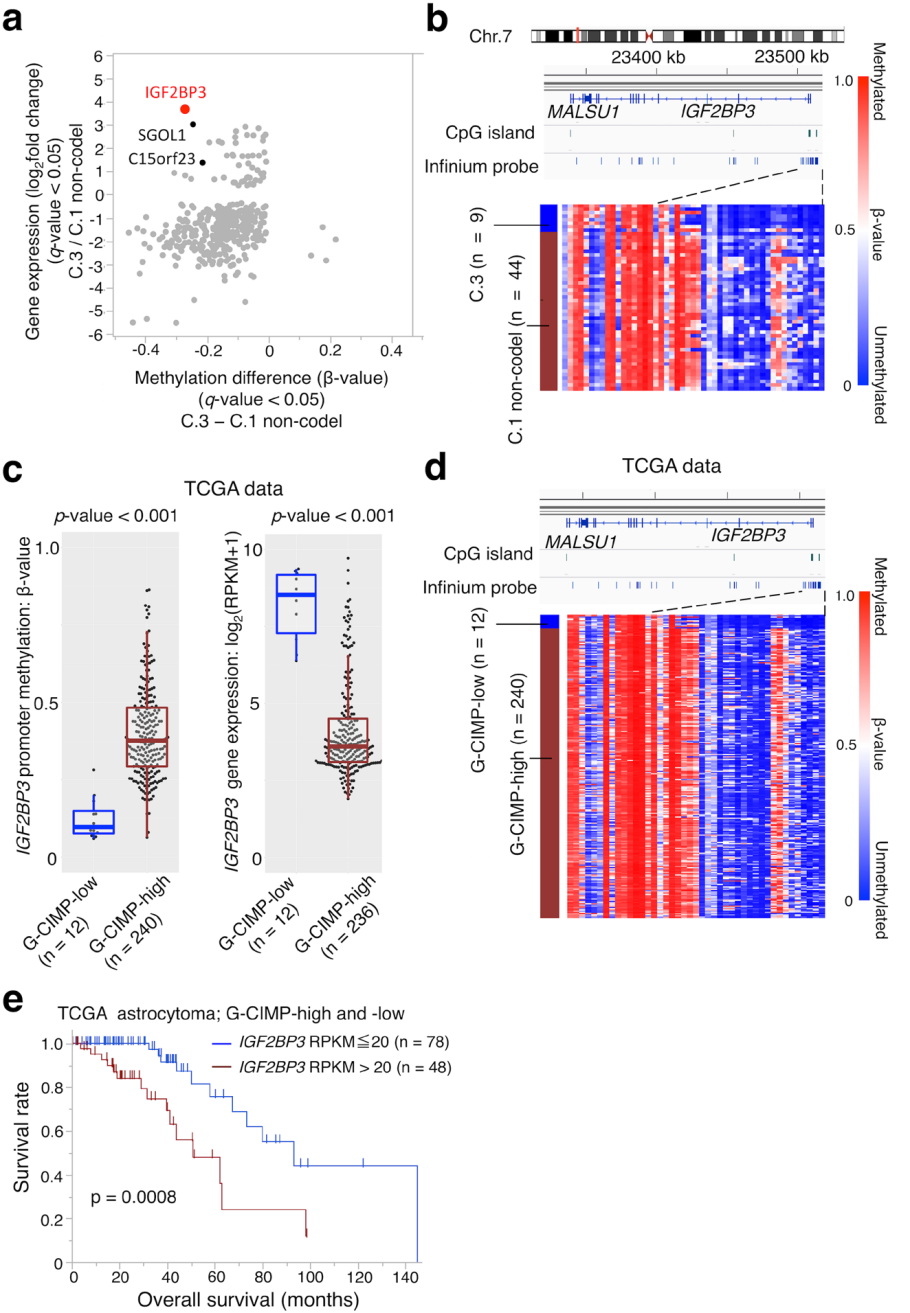
Upregulated genes that showed promoter demethylation. (**a**) A starburst plot comparing gene expression with promoter methylation in G-CIMP-demethylated (C.3) vs. C.1 non-codel tumors is shown. One dot represents one gene. The *q*-values for gene expression and promoter methylation were calculated using a paired two-sided moderated Welch’s *t*-test and the Benjamini-Hochberg method. Genes were considered to be upregulated with promoter demethylation (red dots) when the fold change in gene expression was >2 and methylation difference <−0.2. (**b**) Methylation level of *IGF2BP3* in C.3 and C.1 non-codel tumors. A map of the chromosome around *IGF2BP3* and the positions of methylation array probes are shown at the top. A heatmap of the methylation level of each probe is shown (bottom). Each row represents a sample, and each vertical bar represents a probe. (**c**) Box plots of the *IGF2BP3* promoter methylation level and gene expression comparing G-CIMP-low tumors and G-CIMP-high tumors from the TCGA study. The Wilcoxon rank-sum test was used for statistical analysis. RPKM: reads per kilobase of exon per million mapped sequence reads. (**d**) Methylation level of *IGF2BP3* in G-CIMP-low and G-CIMP-high tumors from the TCGA study is shown, similar to b. (**e**) Kaplan-Meier analysis of overall survival for *IGF2BP3*-high and -low expression tumors among G-CIMP-high and -low tumors in TCGA data. The log-rank test was used for statistical analysis.

To identify genes in which expression was presumably upregulated by demethylation of their promoter region in G-CIMP-demethylated (C.3) tumors, we analyzed changes in gene expression and DNA methylation between C.3 and C.1 non-codel tumors. Infinium probes with annotation of “TSS1500”, “TSS200”, “5′UTR”, or “1stExon” were considered to be located within the promoter, and the average of the top 25% of the most different probes between the two groups in each promoter was calculated to represent the promoter methylation status of the corresponding gene. When criteria to determine that genes were differentially methylated and expressed between two groups were set to 1) *q*-value for both promoter methylation and gene expression <0.05, 2) promoter methylation difference >0.2, and 3) gene expression fold change >2, only three genes were identified that showed upregulated expression and promoter demethylation in C.1 tumors (Fig. 4a,b). The number of identified genes was small probably because most demethylated probes in C.3 were located at non-CpG islands. Nonetheless, these few genes included *IGF2BP3*, which was reported to contribute to cell proliferation in gliomas in a previous report^23^.

To evaluate the significance of *IGF2BP3* in G-CIMP gliomas, the promoter methylation and expression level of this gene were examined using TCGA data, which also include both G-CIMP-high and G-CIMP-low tumors^16^. In agreement with our result, the G-CIMP-low group showed significantly lower methylation in the *IGF2BP3* promoter (p < 0.001) and higher gene expression (p < 0.001) compared with the G-CIMP-high group (Fig. 4c,d). Survival analysis using TCGA data of G-CIMP-high and G-CIMP-low astrocytomas demonstrated that higher expression of *IGF2BP3* was significantly correlated with worse survival of patients (Fig. 4e)^3^. A similar trend was also obtained when *IGF2BP3*-high and -low tumors were compared, even in G-CIMP-high tumors (Supplementary Fig. S4). These results suggested that promoter DNA demethylation and upregulation of *IGF2BP3* may be involved in malignant progression of G-CIMP gliomas.

Instead of using the average of multiple probes in the promoter region, we also compared the raw β-value of each probe between the two groups. In this analysis, the promoter regions of 116 upregulated genes in C.3 tumors compared to C.1 non-codel tumors were analyzed (Supplementary Table S3), and in addition to *IGF2BP3*, the promoters of *TTK*, *CDK2*, and *NCAPG*, all of which are associated with the cell cycle, were shown to have demethylated probes (Supplementary Fig. S5a,b).

### Difference in genetic alterations between initial and progressed gliomas

**Figure 5:**
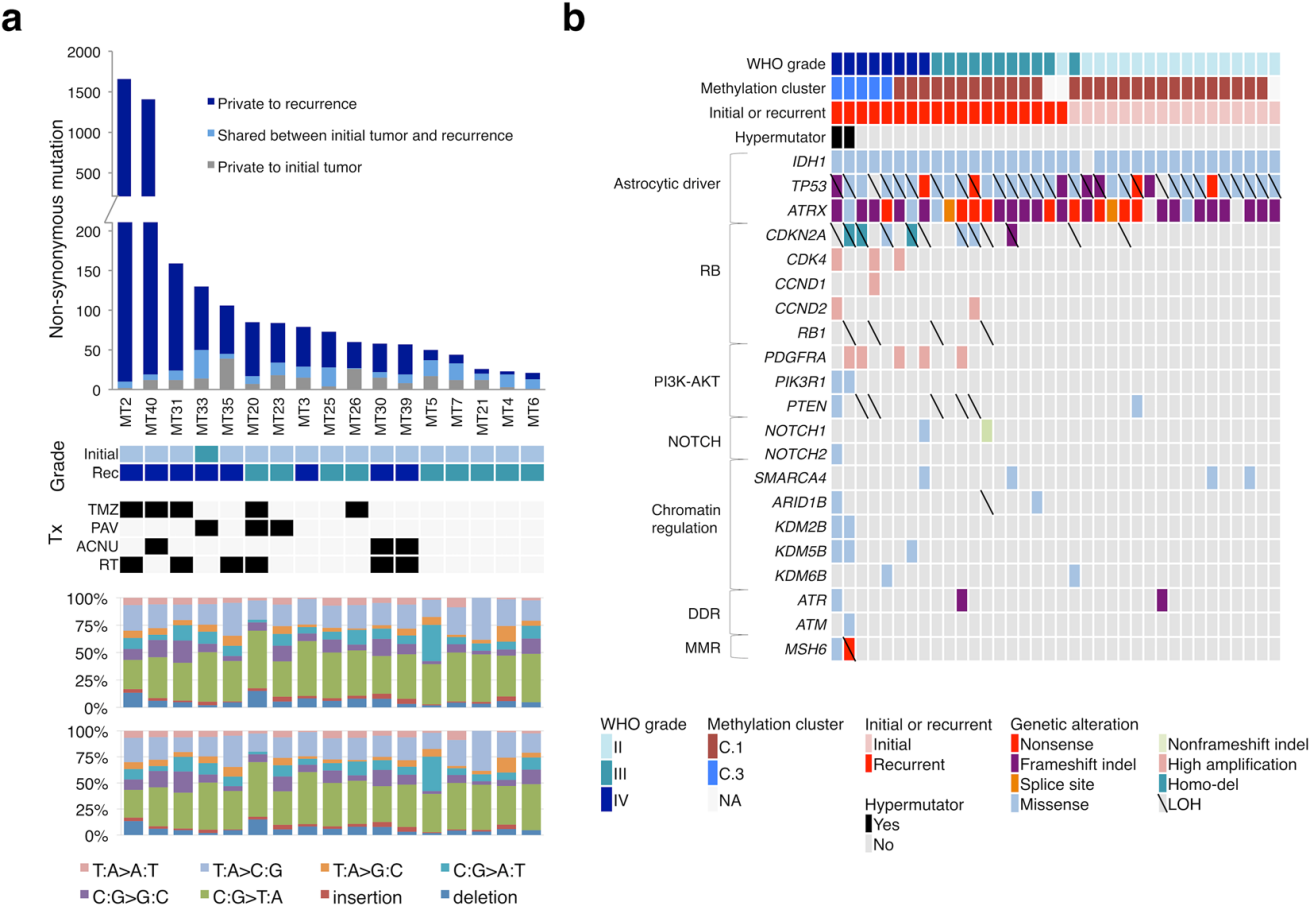
Genomic profile of 17 initial gliomas and 19 corresponding recurrent tumors. (**a**) The numbers of non-synonymous mutations in initial tumors and recurrent tumors in each case are shown at the top. When one case had two recurrences (MT2 and MT20), the second recurrence date is shown. WHO grade of initial and recurrent tumors and treatments performed after the first operation are shown in the middle. The mutation signatures are shown at the bottom. *Tx* treatment, *TMZ* temozolomide, *PAV* procarbazine, nimustine, and vincristine, *ACNU* nimustine, *RT* radiotherapy. (**b**) Representative cancer-related non-synonymous mutations and CNAs of 17 initial tumors and 19 recurrent tumors are shown. Sample information is shown at the top. The types of alterations are indicated as colored boxes. *Indel* insertion and deletion.

Data from whole-exome sequencing (WES) for initial and recurrent paired tumors (17 initial tumors, 17 first recurrent tumors, and two second recurrent tumors) from 17 patients and matched normal blood samples as well as multi-sampling specimens were analyzed (Supplementary Tables S1, 4, 5 and 6). Two recurrent tumors treated with temozolomide (TMZ) had an enormous number of somatic mutations with a characteristic signature and were considered to be TMZ-induced hypermutators (Fig. 5a)^13^. The average numbers of mutations in initial tumors and recurrent non-hypermutator tumors were 26.2 and 56.9, with an average shared mutation rate of 54%. Other driver mutations besides *IDH1*, *TP53*, and *ATRX* were infrequent in initial tumors. In contrast, additional mutations or copy number alterations (CNAs) leading to RB pathway and phosphatidylinositol-3-kinase (PI3K) -AKT pathway dysregulation were frequent in recurrent gliomas, especially in the G-CIMP-demethylated (C.3) tumors. Two TMZ-induced hypermutators were also clustered in C.3 (Fig. 5b). Two spatially separated specimens that showed high or low uptake in m-PET in one glioma tissue both harbored the same *TP53* and *ATRX* mutations, which were considered to be trunk mutations of IDH-mutant astrocytomas (Supplementary Fig. S6a-c). Among mutations specific to the m-PET-high tumor were a gain of function mutation of *PIK3CA* (T1258C) and a truncating mutation of *RB1* (splice site, chromosome 13: 49047536 del). These changes occurred in addition to loss of heterozygosity of this gene locus that was also found in the m-PET-low tissue and led to biallelic *RB1* inactivation (Supplementary Fig. S6c). This m-PET-high tumor with additional driver mutations showed the G-CIMP-demethylated pattern in methylation profile analysis, possibly because of its relatively increased cell proliferation.

Genes encoding enzymes that can directly modify DNA methylation such as *TET* family genes and *DNMT1/3* were not altered in C.3 gliomas despite their remarkable demethylation.

### A novel *MKLN1-MET* fusion was identified in a glioma with the G-CIMP-demethylated profile

**Figure 6:**
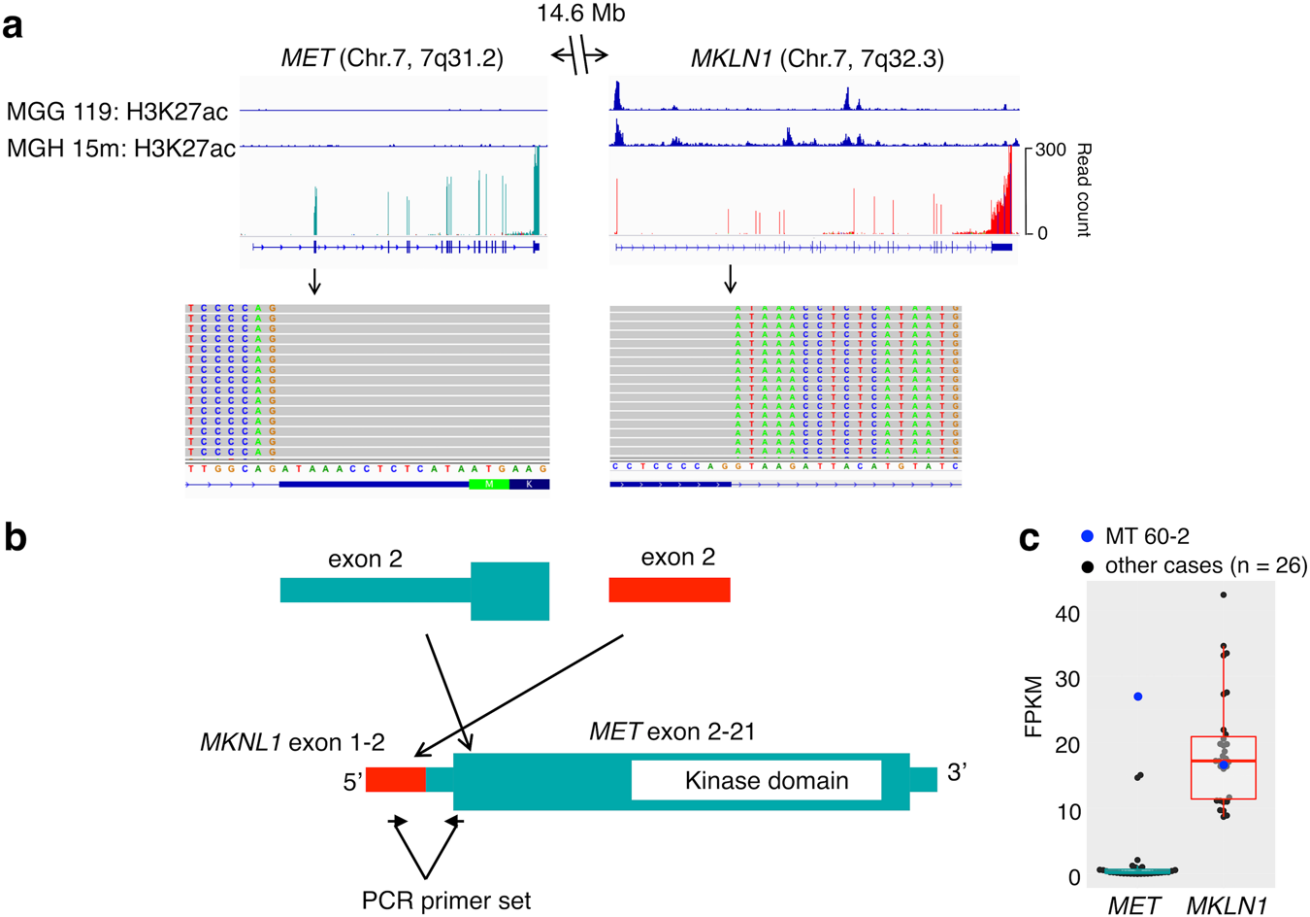
Detection of a novel *MKLN1*-*MET* fusion and the variation. (**a**) Published H3K27ac peak data of an IDH-mutant cell line (MGG119) and tissue (AA15m) are shown (top). Read counts in each exon of *MET* and *MKLN1* of MT 60-2 are shown (middle). Split-reads are shown with alignment on *MKLN* exon 2 and *MET* exon 2 (bottom). (**b**) Schematic of the *MKLN1-MET* fusion transcript. Wide bar indicates the coding sequence, and the narrow bar indicates the untranslated region. Positions of the PCR primer set to specifically amplify the fusion transcript are shown. (**c**) *MET* and *MKLN1* expression levels in MT 60-2 (blue dots) and other samples (black dots) are shown in a dot and box plot.

A novel *MET* fusion was identified in an IDH-mutant recurrent glioma (MT 60-2) in the G-CIMP-demethylated (C.3) group with RNA-seq and was confirmed with direct sequencing following PCR amplification of the cDNA (Fig. 6a, Supplementary Fig. S7). Sequencing reads indicated that a fusion gene was composed of exon 1-2 of *MKLN1* as the 5′-portion and exon 2–21 of *MET* as the 3′-portion (Fig. 6a,b). In fact, many RNA-seq reads of MT 60-2 were mapped in *MET* exon 2–21, but not in exon 1 (Fig. 6a). Because the translation start sites of *MET* and an isoform of *MKLN1* are in exon 2 and exon 6, respectively, we expected that the fusion transcript is translated to the full-length MET protein (Fig. 6b), and that expression of this fusion transcript is regulated by the *MKLN1* promoter. Because *MKLN1*, which encodes the muskelin protein, was expressed by all samples in this study (Fig. 6c), the *MKLN1* promoter is generally active in glioma tissues. Previously reported Histone H3 lysine 27 acetylation (H3K27) ChIP-sequencing (ChIP-seq) data of an IDH-mutant cell line and tumor tissue also showed peaks associated with an active promoter region around the transcription start site of *MKNL1*, but not around that of *MET* (Fig. 6b). Therefore, even though *MET* expression was low in most samples, the fusion gene that may produce the MET protein was highly expressed in this recurrent glioma (Fig. 6c). In other words, *MET* overexpression in this glioma with the G-CIMP-demethylated (C3) profile was possibly driven by the active *MKLN1* promoter created by gene fusion, which led to activation of mitogen-activated protein kinase (MAPK) signaling. This novel *MET* fusion identified in a C.3 tumor may also increase the cell division rate.

## Discussion

Our molecular profile analysis focusing on the differences between initial lower-grade gliomas and recurrent progressed tumors demonstrated that changes observed during malignant transformation are not uniform. However, we found several noticeable characteristics shared by some recurrent tumors. In particular, in accordance with previous observations in the TCGA study, genome-wide DNA methylation analysis demonstrated that a subset of IDH-mutant lower-grade gliomas harboring G-CIMP underwent characteristic CpG locus demethylation during malignant transformation.

While two recent studies by Souza *et al*. and by the TCGA group demonstrated that G-CIMP-low gliomas had worse prognosis than G-CIMP-high gliomas clinically^17,24^, the biological significance of demethylation during malignant progression is unknown. We considered that underlying mechanisms that are associated with this global demethylation must be present, because clustering analysis showed that loci of demethylated probes were generally shared by G-CIMP-demethylated gliomas. Then, we asked whether this demethylation is a cause or a consequence of malignant progression. We expected that DNA demethylation at promoter CpG islands would drive overexpression of several growth-related genes and mediate accelerated tumor growth. However, most demethylation occurred in non-regulatory elements, and the numbers of upregulated genes were limited. We also expected that expression changes or alterations in genes that are responsible for epigenetic modulation would cause massive DNA demethylation; however, no such changes were observed.

Then, we noticed an interesting finding in which estimated late-replicating chromatin regions were more demethylated than early-replicating regions. Whereas DNMT3A and DNMT3B establish *de novo* methylation patterns, maintenance of DNA methylation during cell division is usually achieved by the activity of DNMT1, which recognizes hemimethylated sites and replicates the methylation patterns^25^. If this enzyme does not work properly and sufficiently, gradual loss of methylation occurs during cell division. Such a mechanism of demethylation is called “passive demethylation”, in contrast to “active demethylation” caused by demethylation factors such as TET family members. Recent reports showed that loss of DNA methylation in cancers compared with adjacent normal tissue often occurs in late-replicating, nuclear lamina-associated domains and that such demethylation is more remarkable in tumors with higher expression of cell cycle-related genes^17,18^. Another study using normal cell lines demonstrated that late-replicating regions are gradually demethylated as the cell divides, in contrast to early-replicating regions in which methylation is better maintained^20^. These data indicated that loss of methylation in late-replicating domains reflects mitotic activity. These hypotheses are also applicable in the context of malignant progression of gliomas; regions that were markedly demethylated in recurrent tumors that showed accelerated cell division were likely to be located at late-replicating regions. Furthermore, this finding was verified with the TCGA data set, suggesting that most demethylation that occurs in progressed tumors may be “passive demethylation” that happens during accelerated cell division. We also demonstrated that IDH1-mutant cell lines exhibited a demethylation profile. Cell lines tend to lose methylation preferentially in late-replicating regions, because primary cultured cells generally divide more rapidly than cells in original tumors, and thus, maintenance of methylation cannot keep up. The fact that both IDH1-mutant cell lines and recurrent tumor samples showed a similar hypomethylated profile further supports the idea that “passive demethylation” actually plays a role during tumor progression.

Although most DNA demethylation, which we think occurred in a passive manner as a consequence of tumor progression, did not increase gene expression, we identified a few genes that were transcriptionally upregulated with CpG locus demethylation such as *IGF2BP3*. IGF2BP3 is an RNA binding protein that binds to the 5′-untranslated region of *IGF-2* mRNA and activates its translation^26^. Suvasini *et al*. showed that *IGF2BP3* is upregulated especially in GBMs^23^. They demonstrated that IGF2BP3 increases the IGF-2 protein level without increasing its transcript level, and promotes tumor growth by activating downstream effectors such as PI3K and MAPK. Dutoit *et al*. also showed overexpression of *IGF2BP3* in astrocytomas and GBMs^27^ and that its expression level was increased in six of seven recurrent secondary GBMs that progressed from grade II or III astrocytomas. Although no mechanism of *IGF2BP3* upregulation was shown in previous studies, our data indicated that promoter demethylation causes gene upregulation during malignant progression^16^. *IGF2BP3* may be associated with malignant progression of IDH1-mutant gliomas and be a candidate therapeutic target. As previous reports also reported that other upregulated cell cycle-related genes show promoter demethylation besides *IGF2BP3*, DNA demethylation may be part of the mechanism that promotes tumor progression^14^.

Another hypothesis is that loss of DNA methylation activates some enhancers of key growth-regulating genes that are associated with malignant progression. Thus, we examined whether demethylation occurred in enhancer regions that may have been identified by the H3K27ac peak in the ChIP-seq data of the IDH-mutant cell line (MGG119). However, DNA demethylation around enhancer regions was not identified for 116 genes that were upregulated in G-CIMP-demethylated tumors (data not shown). However, the methylation array analysis that we performed in this study is insufficient to evaluate the enhancer status because the number of probe positions is limited. Further studies with whole-genome bisulfite sequencing will be needed to determine whether DNA demethylation at enhancer regions plays a role in malignant progression.

Our data also demonstrated several other mechanisms that possibly enhanced the rate of cell division during malignant transformation. In accordance with previous reports^16^, mutations and CNAs in genes involved in the RB and PI3K-AKT pathways were frequent in recurrent tumors; such alterations were especially frequent in G-CIMP-demethylated tumors. In addition to such known findings, a novel *MET* fusion was identified in a demethylated tumor. Although a few types of *MET* fusions have already been reported, the *MKLN1-MET* fusion is novel to the best of our knowledge^28,29^. In our case, *MET* was upregulated by hijacking the active *MKLN1* promoter. Interestingly, Bao *et al*. showed that the *PTPRZ-MET* fusion was also frequent in IDH-mutant GBMs^29^. Thus, we suggest that in addition to mutations and CNAs, these fusions could cause upregulation of cell cycle and cell growth-related genes that are associated with malignant transformation and could be druggable targets^17,24,28^.

**Figure 7:**
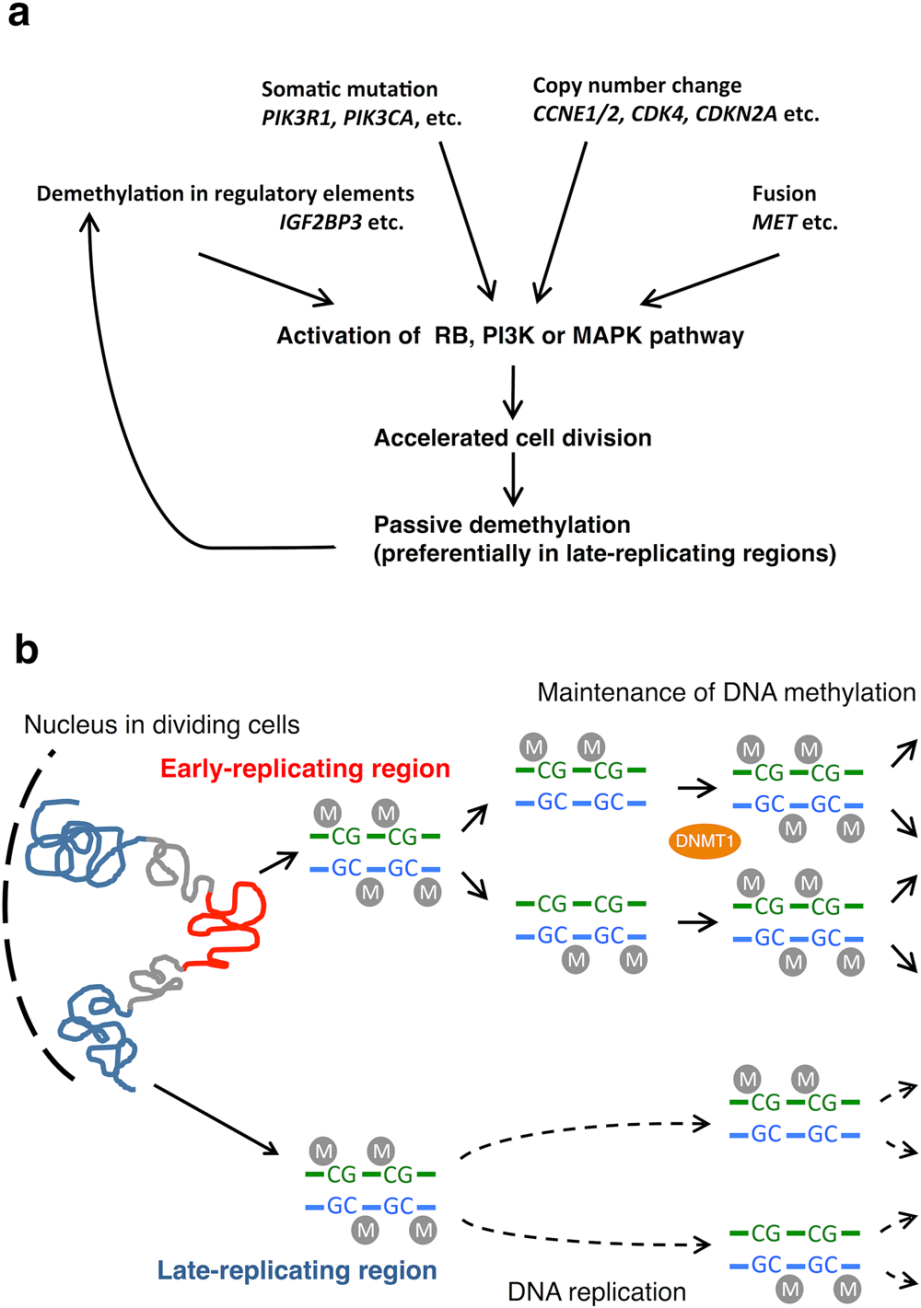
Schematic overview of DNA demethylation during glioma progression. (**a**) Somatic mutations, CNAs, genomic fusion, and demethylation in the promoter and enhancer of RB, PI3K-AKT or MAPK pathway genes upregulate cell cycle and cell growth-related genes, which accelerate cell division and lead to passive demethylation preferentially in late-replicating regions. Upregulated genes by the demethylation such as *IGF2BP3* further accelerates cell division. (**b**) When DNA replicates, the methylation pattern is maintained by DNA methyltransferase (DNMT1) by copying it to daughter strands. However, in vigorously dividing cells, passive demethylation may occur at late-replicating regions due delayed maintenance of methylation.

In Summary, we demonstrated that as previously reported, global demethylation after malignant progression in a subset of G-CIMP gliomas was also evident in our set of recurrent astrocytic tumors. We concluded that most demethylation events are likely due to “passive demethylation” preferentially in late-replicating regions following accelerated cell division during malignant transformation caused by genomic and transcriptome changes, although some loss of methylation including that in the *IGF2BP3* promoter could be related to tumor progression. Such molecules potentially accelerate tumor progression and thus may be candidate therapeutic targets (Fig. 7a,b).

## Materials and Methods

### Patients and clinical samples

Clinical samples were obtained from patients who provided informed consent and who underwent surgery at The University of Tokyo Hospital, National Cancer Center Hospital, Kyorin University Hospital, Dokkyo Medical University Hospital, or Saitama Medical University International Medical Center. This study was approved by the ethics committees of each institute. Histological diagnoses were made by an experienced neuropathologist at each hospital.

In total, we used 125 glioma samples, including 99 IDH-mutant and 26 IDH-wildtype tumors. Of 99 IDH-mutant tumors, 30 tumors (20 oligodendrogliomas and 10 anaplastic oligodendrogliomas) had the codel, and 69 tumors (37 diffuse astrocytomas, 17 anaplastic astrocytomas, and 15 GBMs) did not have the codel. Of these 69 IDH-mutant tumors without the codel, 24 matched pairs of initial lower-grade astrocytic tumors and recurrent tumors were included. This data set also included tumors from a patient who showed intratumoral malignant transformation (initial lower-grade and higher-grade portions in a single resected tumor). Detailed information of the samples used in this study is provided in Supplementary Table S1.

### DNA and RNA preparation

The Allprep DNA/RNA Micro Kit (Qiagen) was used to extract genomic DNA and RNA samples from freshly frozen tumor tissues. The QIAamp DNA Mini Kit (Qiagen) was used to prepare genomic DNA samples from normal blood. A Qubit Assay Kit (Thermo Fisher Scientific) was used to measure the concentration of double-stranded DNA, and the 2100 Bioanalyzer system (Agilent Technologies) was used to measure the quality of RNA; the RNA Integrity Number was more than 7 in most RNA samples. All kits were used according to the manufacturers’ protocols.

### Analysis of common molecular alterations in gliomas

Sanger sequencing was performed to detect the hotspot mutation of *IDH1* and *IDH2*, and microsatellite analysis was used to detect loss of heterozygosity on chromosome (chr.) 1p and 19q as previously described^30^. When tumors had no available paired blood DNA, multiplex ligation-dependent probe amplification (MLPA) was performed using the ASALSA MLPA kit P088 (MRC Holland) according to the manufacturer’s instructions. *IDH1* and 1p/19q status are shown in Supplementary Table S1.

### Genome-wide methylation profiling

The Infinium HumanMethylation450K BeadChip (Illumina) was used to analyze the genome-wide methylation profile of 122 gliomas according to the manufacturer’s instructions, as previously described (Supplementary Table S1)^31,32^. Data for our nine previously reported IDH-mutant tumors with the codel were used in this study^32^. For each CpG site, the ratio of the fluorescent signal (β-value) was calculated by comparing the signal from a methylated probe relative to the sum of signals from methylated and unmethylated probes. This β-value, ranging from 0.00 to 1.00, reflected the methylation level of the individual CpG site represented by the probe. The following filtering steps were used to select probes for unsupervised clustering analysis. Probes targeting the X and Y chromosomes and probes associated with a single nucleotide polymorphism (dbSNP 130 common) within five base pairs of and including the targeted CpG site were excluded^7,33^. For clustering analysis of 122 samples, the standard deviation of β-values for each probe was calculated, and the top 10,000 most variable probes were selected. Unsupervised clustering analysis using a Euclidean distance and the ward.D2 linkage method was performed.

### DNA methylation and replication timing

Genome-wide replication timing data from NPCs  were obtained from GSE63428 and comparatively analyzed with DNA methylation data that we obtained from our samples using the Infinium HumanMethylation450K BeadChip^19^. Each Infinium probe was annotated to late replication, intermediate replication, or early replication according to the replication timing value at each Infinium probe position (late replication, replication timing value ≤ −0.4599; intermediate replication, −0.4599 < replication timing value ≤ 0.80074; early replication, 0.80074 < replication timing value). The thresholds −0.4599 and 0.80074 were determined by calculating the median of the negative and positive replication timing value, respectively. The distribution of Infinium probes according to replication timing was statistically analyzed using the Chi-square test.

### Gene expression analysis

As previously described^31^, RNA-seq was performed for 31 samples that had RNA of sufficient quality and quantity (Supplementary Table S1). An RNA-seq library was prepared using the TruSeq Stranded mRNA LT Sample Prep Kit (Illumina) according to the manufacturer’s protocol. Briefly, 1 µg total RNA was purified using oligo dT magnetic beads, and polyA+ RNA was fragmented at 94 °C for 2 min. cDNA was synthesized using Superscript II (Invitrogen), and adapter-ligated cDNA was amplified with 12-cycle PCR. Each library was sequenced using HiSeq2000 (Illumina) by loading four libraries per lane of the flow cell, producing an average of 30.6 million pairs of 101-cycle reads for each sample. NGS reads are mapped to a human cDNA database (UCSC genes) and the reference genome GRCh37/hg19 independently using the Burrows-Wheeler Aligner (BWA). After the cDNA coordinates were converted to genomic positions, an optimal mapping result was chosen either from cDNA or genome mapping by comparing the minimal edit distance to the reference. Then, local realignment was done with an in-house short reads aligner with a small seed size (k = 11) (Qgram-SmithWaterman). Finally, FPKM values were calculated for each UCSC gene, considering strand-specific information.

### Mutation detection and copy number analysis using WES

WES was performed for 38 tumor samples from 18 patients and matched blood samples using the SureSelect Human All Exon Kit (Agilent Technologies) according to the manufacturer’s protocols, as previously described (Supplementary Table S1)^31,34,35^. Data for our 13 previously reported tumors from six patients were also used in this study^13^. Sequencing was performed with the Illumina Hiseq2000 as 100-bp paired-end reads. Sequencing data are summarized in Supplementary Table S4. The BWA and NovoAlign software (Novocraft Technologies) were used to align NGS reads to the human reference genome GRCh37/hg19. After removal of PCR duplicates, the Short-Read Micro re-Aligner was used to improve variant discovery through local realignments. Somatic mutations, CNAs, and tumor purity were detected using integrated genotyper software (karkinos: http://github.com/genome-rcast/karkinos) as previously reported (Supplementary Tables S5 and 6).

### Fusion transcript detection and validation

Fusion analysis was performed as previously described using Genomon-fusion (https://genomon-project.github.io/GenomonPagesR/) with default parameters^31^. At least 12 bases matching both sides of the fusion in each read and more than four reads spanning the candidate break point were required to call the fusion transcript. When two sides resided on the same chromosome, we chose a minimum distance of 100,000 bp to reduce read-through transcripts. To validate *MKLN1-MET* fusion transcripts, tumor RNA was reverse-transcribed using Superscript III (Invitrogen) according to the manufacturer’s protocol, and the obtained cDNAs were used as the PCR template. Oligo primers for PCR were designed in exon 1 of *MKLN1* (5′-TACGCGCTACACAAGTGGAG-3′) and exon 2 of *MET* (5′-CACTCCCCATTGCTCCTCTG-3′) to amplify only the fusion transcript. PCR was performed using KOD-Plus-NEO (Toyobo) with optimized thermal conditions. PCR products were sequenced to confirm the presence of the fusion product.

### Statistical analysis

To identify differentially methylated probes or differentially expressed genes between two groups, Welch’s *t*-test and the Benjamini-Hochberg method were used to calculate *p*-values and *q*-values, respectively.

### Public data acquisition

The Infinium HumanMethylation450K BeadChip data and RNA-seq data of TCGA projects were obtained from the website (https://tcga-data.nci.nih.gov). The Infinium HumanMethylation450K BeadChip data of two IDH-mutant cell lines (MGG119 and MGG152) were obtained from the Gene Expression Omnibus (GEO, http://www.ncbi.nlm.nih.gov/go) of the National Center for Biotechnology Information (NCBI) and were accessed through GEO series accession number GSE 73270^31^. Histone H3 lysine 27 acetylation ChIP-seq data of an IDH-mutant cell line (MGG119) and tissue (AA15m) were obtained from GSE70991^36^.

### Ethics approval and informed consent

All procedures performed in studies involving human participants were in accordance with the ethical standards of the institutional and with the 1964 Helsinki declaration and its later amendments or comparable ethical standards. This study was approved by the research ethics committees of The University of Tokyo (No. G10028) and other institutes. Informed consent was obtained from all individual participants included in the study.

## Supplementary information

Supplementary figure

Supplementary Table S1-S6

## Data Availability

The datasets generated and analyzed during the current study are available in Japanese Genotype-phenotype Archive (JGA, http://trace.ddbj.nig.ac.jp/jga) under accession number JGAS00000000146.
