# Publisher Correction: DNA demethylation is associated with malignant progression of lower-grade gliomas

**DOI:** 10.1038/s41598-019-43790-7

**Published:** 2019-05-23

**Authors:** Masashi Nomura, Kuniaki Saito, Koki Aihara, Genta Nagae, Shogo Yamamoto, Kenji Tatsuno, Hiroki Ueda, Shiro Fukuda, Takayoshi Umeda, Shota Tanaka, Shunsaku Takayanagi, Ryohei Otani, Takahide Nejo, Taijun Hana, Satoshi Takahashi, Yosuke Kitagawa, Mayu Omata, Fumi Higuchi, Taishi Nakamura, Yoshihiro Muragaki, Yoshitaka Narita, Motoo Nagane, Ryo Nishikawa, Keisuke Ueki, Nobuhito Saito, Hiroyuki Aburatani, Akitake Mukasa

**Affiliations:** 1 Department of Neurosurgery, Graduate School of Medicine, The University of Tokyo, 7-3-1 Hongo, Bunkyo-ku, Tokyo 113-8655 Japan; 2 Genome Science Division, Research Center for Advanced Science and Technology, The University of Tokyo, 4-6-1 Komaba, Meguro-ku, Tokyo 153-8904 Japan; 3 Department of Neurosurgery, Kyorin University Faculty of Medicine, 6-20-2 Shinkawa, Mitaka-shi, Tokyo 181-8611 Japan; 4 Department of Neurosurgery, Cancer and Infectious Disease Center Tokyo Metropolitan Komagome Hospital, 3-18-22 Honkomagome, Bunkyo-ku, Tokyo 113-8677 Japan; 5 Department of Neurosurgery, Dokkyo Medical University, 880 Kitakobayashi, Mibu-machi, Shimotsuga-gun, Tochigi 321-0293 Japan; 6 Department of Neurosurgery, Graduate School of Medicine, Yokohama City University, 3-9 Fukuura, Kanazawa-ku, Yokohama 236-0004 Japan; 7 Department of Neurosurgery, Tokyo Women’s Medical University, 8-1 Kawada-cho, Shinjuku-ku, Tokyo 162-8666 Japan; 8 Department of Neurosurgery and Neuro-Oncology, National Cancer Center Hospital, 5-1-1 Tsukiji, Chuo-ku, Tokyo 104-0045 Japan; 9 Department of Neuro-Oncology/Neurosurgery, Saitama Medical University International Medical Center, 1397-1 Yamane, Hidaka-shi, Saitama 350-1298 Japan; 10 Department of Neurosurgery, Graduate School of Medical Sciences, Kumamoto University, 1-1-1 Honjo, Chuo-ku, Kumamoto 860-8556 Japan

Correction to: *Scientific Reports* 10.1038/s41598-019-38510-0, published online 13 February 2019

In the original version of this Article, Masashi Nomura and Kuniaki Saito were omitted as equally contributing authors. This has now been corrected in the PDF and HTML versions of the paper.

