# Supplementary material for: DNA demethylation is associated with malignant progression of lower-grade gliomas
Source: Sci Rep. 2019 Feb 13;9:1903. doi: 10.1038/s41598-019-38510-0 (PMC6374451; doi:10.1038/s41598-019-38510-0)
Supplement: Supplementary file 1 — Supplementary figure [file 41598_2019_38510_MOESM1_ESM.pdf]

Supplementary Figures

Title:

DNA demethylation is associated with malignant progression of lower-grade gliomas

Authors:

Masashi Nomura, Kuniaki Saito, Koki Aihara, Genta Nagae, Shogo Yamamoto, Kenji Tatsuno, Hiroki Ueda, Shiro Fukuda, Takayoshi Umeda, Shota Tanaka, Shunsaku Takayanagi, Ryohei Otani, Takahide Nejo, Taijun Hana, Satoshi Takahashi, Yosuke Kitagawa, Mayu Omata, Fumi Higuchi, Taishi Nakamura, Yoshihiro Muragaki, Yoshitaka Narita, Motoo Nagane, Ryo Nishikawa, Keisuke Ueki, Nobuhito Saito, Hiroyuki Aburatani and Akitake Mukasa.

Corresponding author:

Akitake Mukasa

Department of Neurosurgery, Graduate School of Medicine, The University of Tokyo

E-mail: mukasa-nsu@umin.ac.jp

Hiroyuki Aburatani

Genome Science Division, Research Center for Advanced Science and Technology (RCAST), The University of Tokyo

E-mail: haburata-tky@umin.ac.jp

Fig. S1 – S7

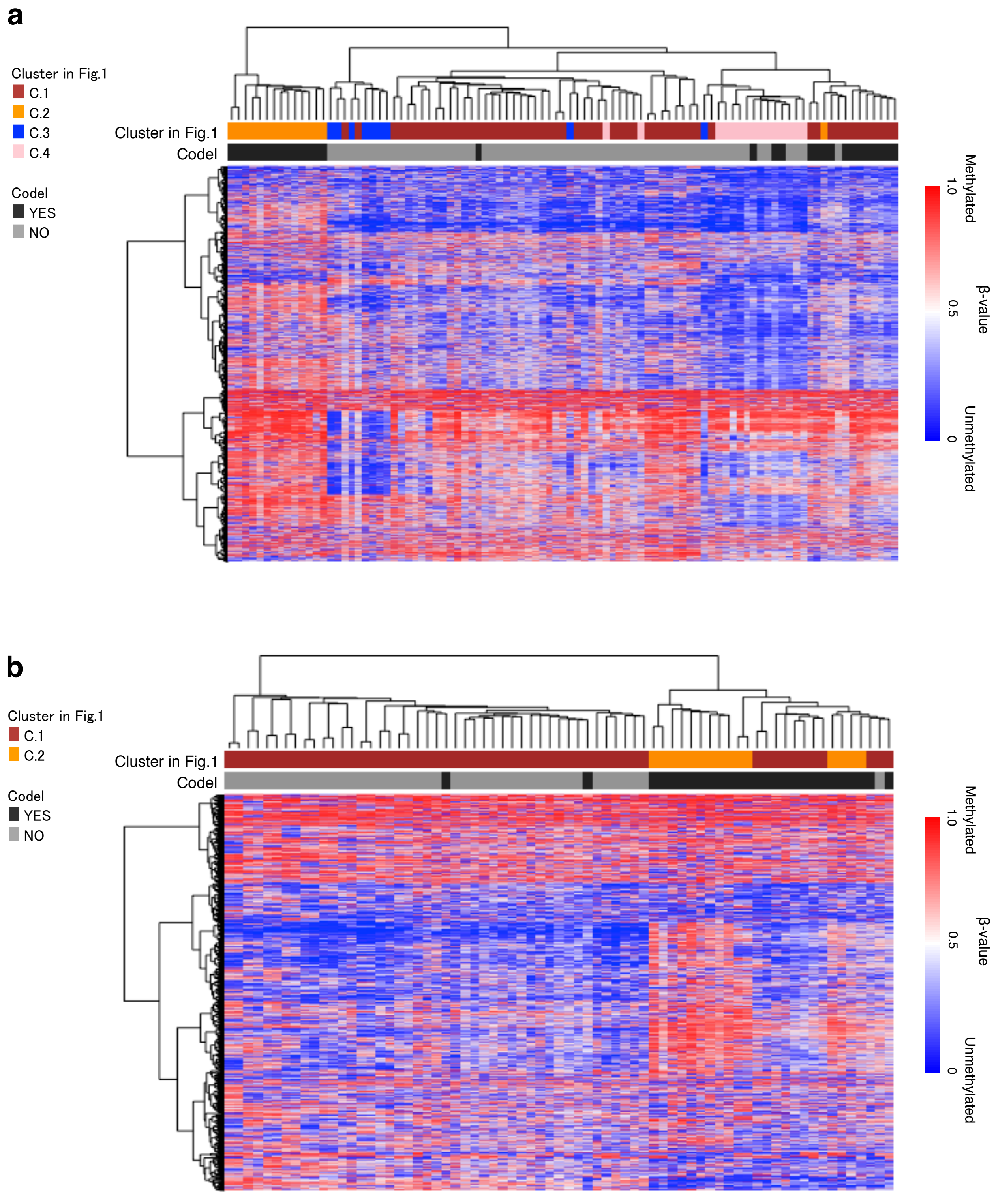

Fig. S1 DNA methylation profile of IDH-mutant gliomas. Unsupervised clustering was performed. The Euclidean distance and the ward.D2 linkage method were used. Sample information is shown at the top. **a** Top 10,000 variant probes of C.1 – C.4 tumors (95 samples) were used. **b** Top 3,000 variant probes of C.1 and C.2 tumors (71 samples) were used

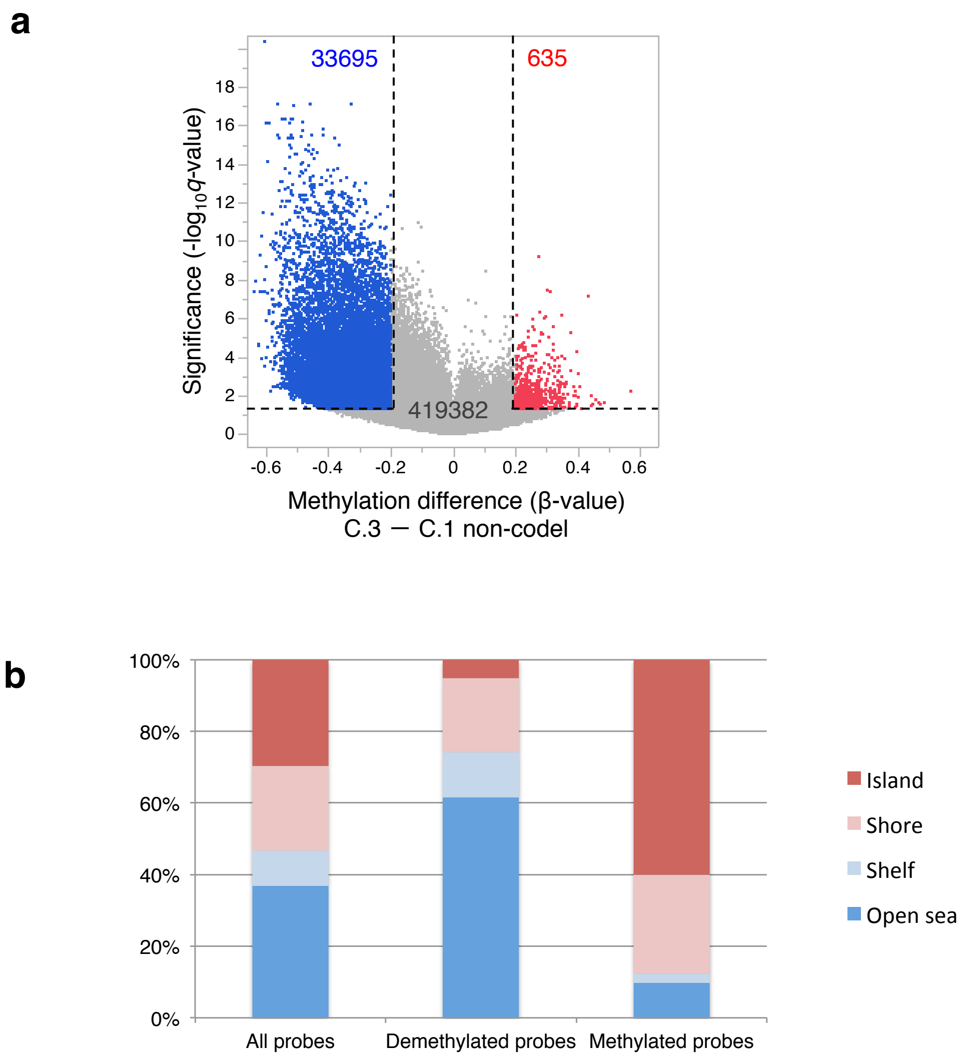

Fig. S2 Distribution of demethylated probes during malignant transformation. **a** A volcano plot comparing the methylation level of each probe between G-CIMP-demethylated (C.3) tumors and C.1 non-codel tumors in this study. One dot represents one probe. The q-values were calculated using a paired two-sided moderated Welch's t-test and the Benjamini-Hochberg method. Probes were considered to be significantly different in C.3 tumors when the q-value was < 0.05 and the methylation difference was > 0.2. **b** Distribution of significantly methylated probes and demethylated probes in C.3 tumors is shown. CpG probes are classified into "CpG island", "shores", "shelf", and "open seas", as defined by the UCSC annotation.

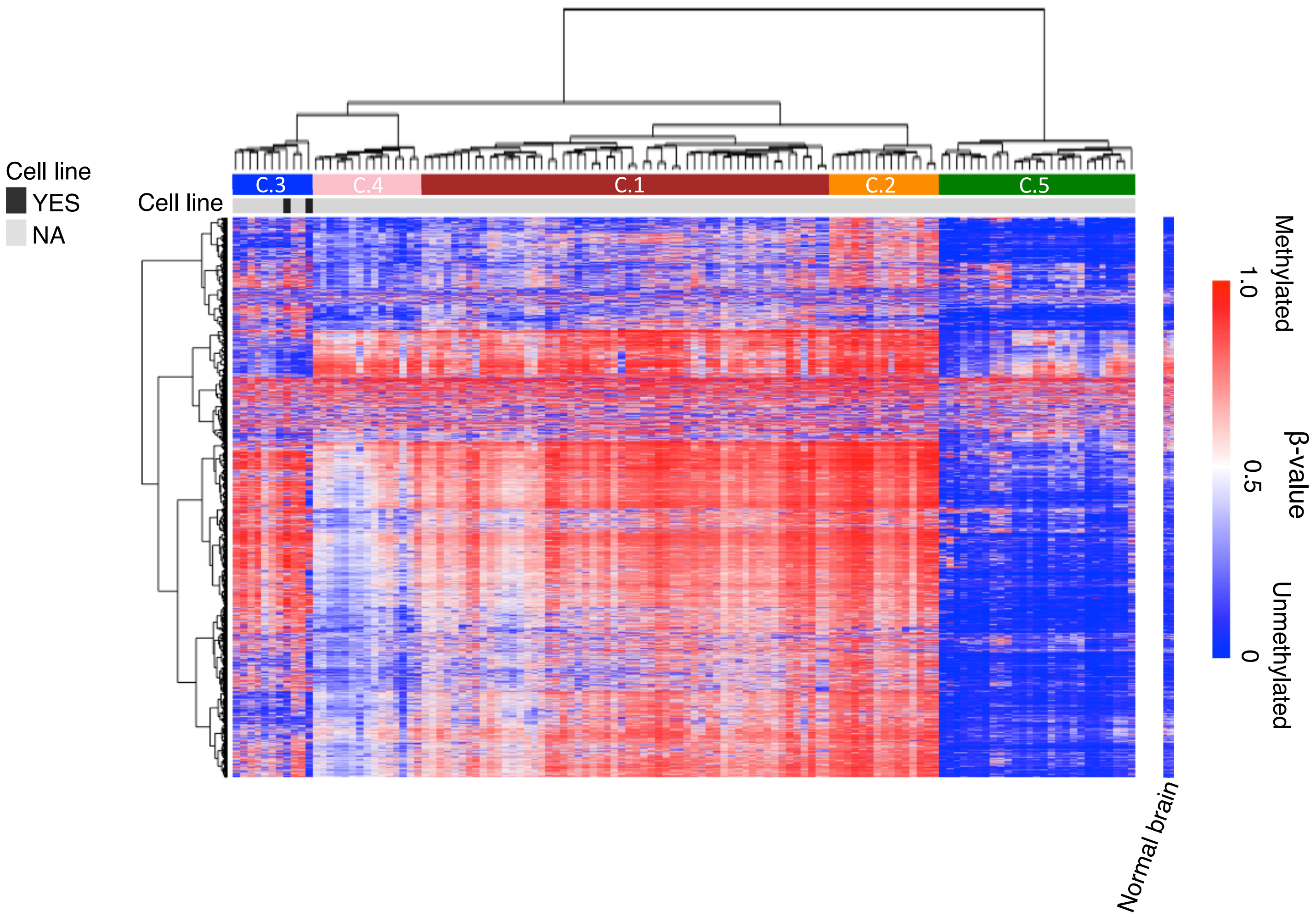

Fig. S3 DNA methylation profile of two IDH1-mutant cell lines and 122 glioma tissues. The Infinium Human Methylation 450K BeadChip data for two IDH-mutant cell lines (MGG119 and MGG152) from a previous report were combined with our data for 122 gliomas, and unsupervised clustering was performed using the top 10,000 probes used in Fig. 1. Heatmap of the DNA methylation profile in the two IDH-mutant cell lines, our 122 gliomas, and three samples of normal brain is shown. Heatmap of three normal brain samples using the same probes with tumor clustering is shown on the right.

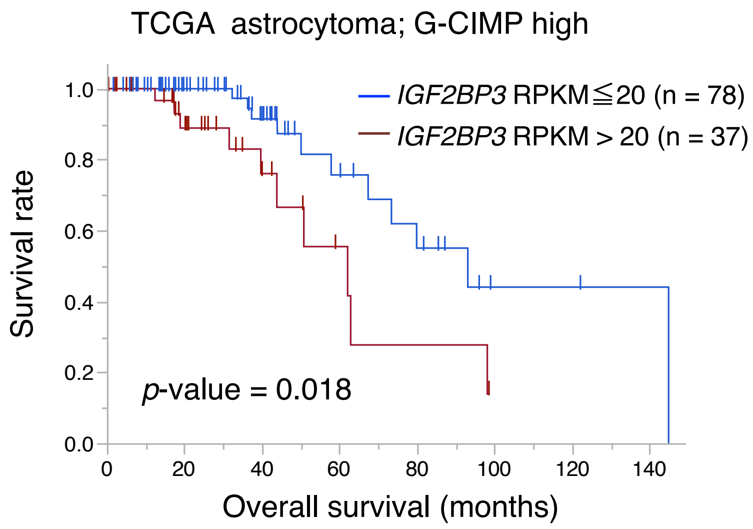

Fig. S4 Kaplan-Meier analysis of overall survival of patients with *IGF2BP3*-high and -low expression tumors among G-CIMP-high tumors in TCGA data. A Kaplan-Meier curve is shown. The log-rank test was used for statistical analysis.

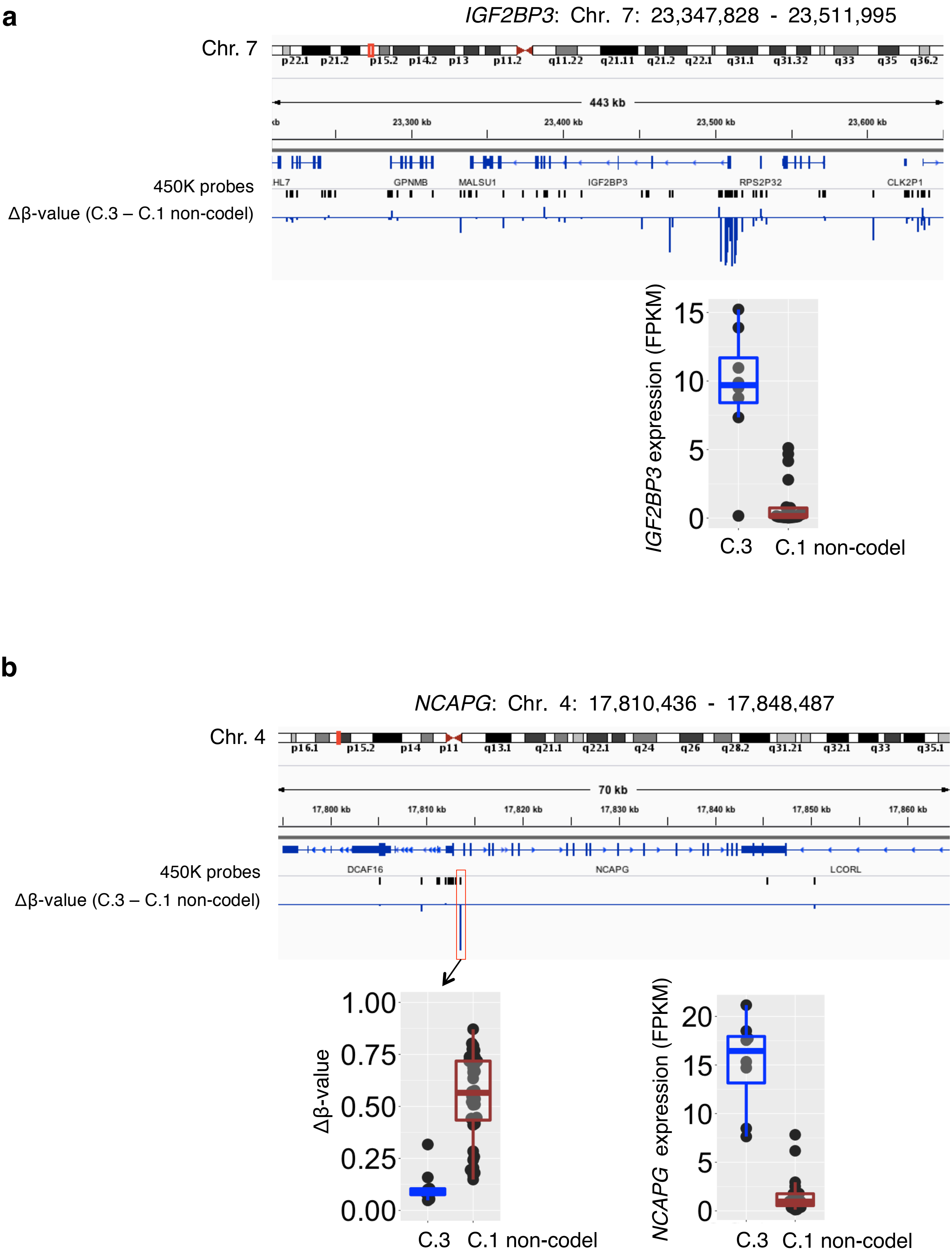
a
IGF2BP3: Chr. 7: 23,347,828 - 23,511,995
Chr. 7
p22.1 p21.2 p15.2 p14.2 p13 p11.2 q11.22 q21.11 q21.2 q22.1 q31.1 q31.32 q33 q35 q36.2
443 kb
23,300 kb 23,400 kb 23,500 kb 23,600 kb
HL7 GPNMB MALSU1 IGF2BP3 RPS2P32 CLK2P1
450K probes
Δβ-value (C.3 – C.1 non-codel)
IGF2BP3 expression (FPKM)
15
10
5
0
C.3
C.1 non-codel
b
NCAPG: Chr. 4: 17,810,436 - 17,848,487
Chr. 4
p16.1 p15.2 p14 p11 q13.1 q21.1 q22.1 q24 q26 q28.2 q31.21 q32.1 q33 q35.1
70 kb
17,800 kb 17,810 kb 17,820 kb 17,830 kb 17,840 kb 17,850 kb 17,860 kb
DCAF16 NCAPG LCORL
450K probes
Δβ-value (C.3 – C.1 non-codel)
Δβ-value
1.00
0.75
0.50
0.25
0.00
C.3
C.1 non-codel
NCAPG expression (FPKM)
20
15
10
5
0
C.3
C.1 non-codel

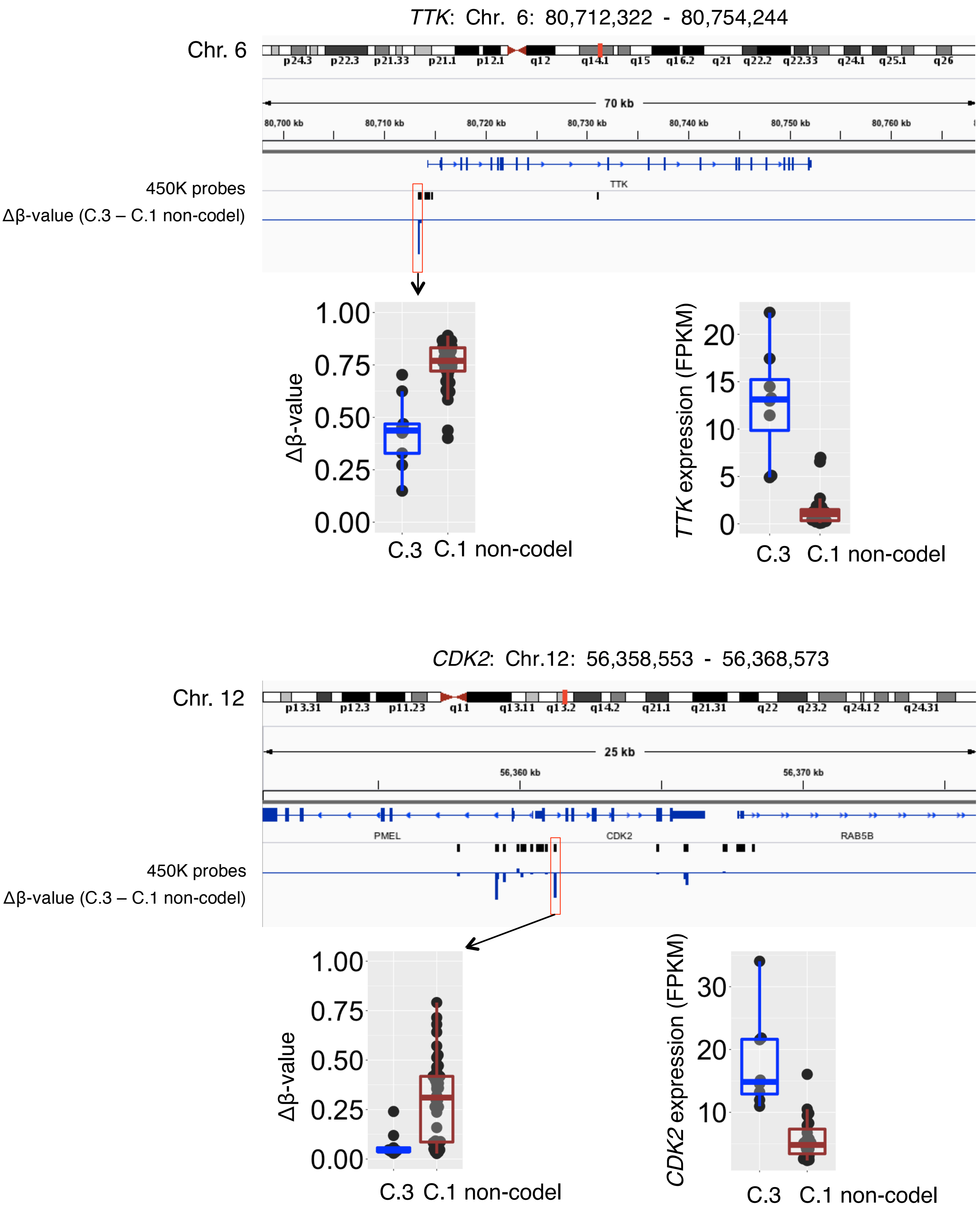

TTK: Chr. 6: 80,712,322 - 80,754,244
Chr. 6
70 kb
TTK
450K probes
Δβ-value (C.3 – C.1 non-codel)
Δβ-value
1.00
0.75
0.50
0.25
0.00
C.3
C.1 non-codel
TTK expression (FPKM)
20
15
10
5
0
C.3
C.1 non-codel
CDK2: Chr.12: 56,358,553 - 56,368,573
Chr. 12
25 kb
56,360 kb
56,370 kb
PMEL
CDK2
RAB5B
450K probes
Δβ-value (C.3 – C.1 non-codel)
Δβ-value
1.00
0.75
0.50
0.25
0.00
C.3
C.1 non-codel
CDK2 expression (FPKM)
30
20
10
C.3
C.1 non-codel

Fig. S5 Loss of DNA methylation around the transcription start site of upregulated genes during malignant transformation. **a** A snapshot of the Integrative Genomics Viewer (IGV) demonstrating positions of methylation array probes and the difference in the β-value (Δβ-value) G-CIMP-demethylated (C.3) and C.1 non-codel tumors at each probe around *IGF2BP3* is shown at the top. A box plot of gene expression (*IGF2BP3*) comparing C.3 and C.1 non-codel tumors is shown below. **b** Snapshots of the IGV viewer showing probe positions of the methylation array and the difference in the β-value (Δβ-value) between C.3 and C.1 non-codel tumors at each probe around *NCAPG*, *TTK*, and *CDK2* are shown at the top. Box plots of the Δβ-value at probes in the red quadrilaterals comparing C.3 and C.1 non-codel tumors are shown on the left. Box plots of gene expression (*NCAPG*, *TTK*, and *CDK2*) comparing C.3 and C.1 non-codel tumors are shown on the right.

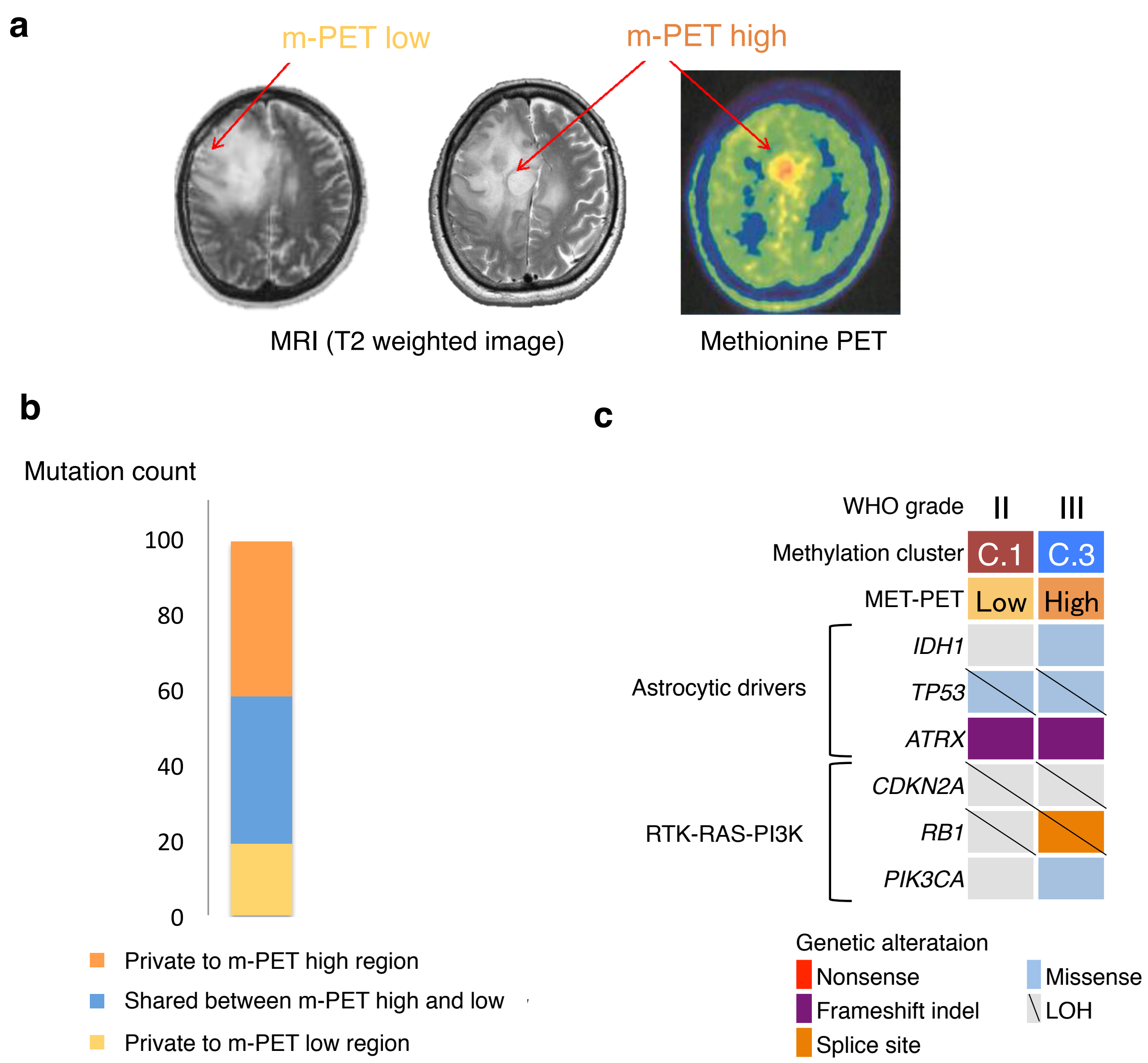

Fig. S6 Analysis of two spatially separated tumor specimens from a patient. **a** This case was diagnosed as a lower-grade glioma on magnetic resonance imaging (MRI) and was followed up. However, during the follow-up, the tumor grew bigger, and an m-PET high lesion emerged. m-PET low (left) and high (right) regions were analyzed. MRI and m-PET findings are shown. Methylation analysis (Fig. 1) showed that a specimen from the peripheral region was clustered in C.1, but a specimen from the core m-PET high specimen was clustered in C.3. **b** The number of non-synonymous mutations of m-PET low and high regions. **c** Representative cancer-related non-synonymous mutations and CNAs of m-PET low and high regions. The types of alterations are indicated as colored boxes. The *IDH1* R132H mutation was also detected in both specimens with Sanger sequencing, although the mutation was not detected in the m-PET-low specimens with WES data analysis, probably due to its low mutant allele frequency (1 of 17 reads) caused by contamination of the analyzed sample with normal tissue.

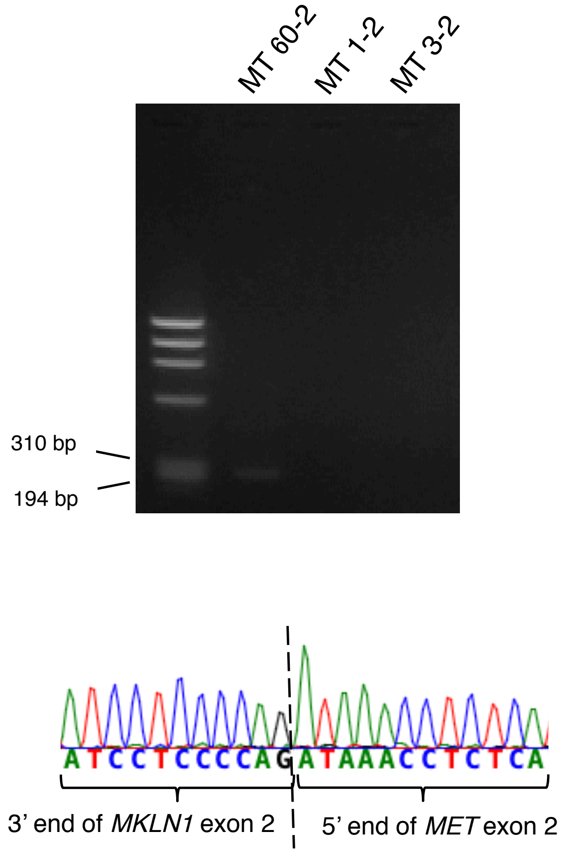

Fig. S7 Validation of the *MKLN1-MET* fusion by PCR and Sanger sequencing. The estimated PCR band (208 bp) was detected specifically in MT 60-2. The predicted sequence was confirmed by Sanger sequencing.
